# Loss of H2A.Z Is Not Sufficient to Determine Transcriptional Activity of Snf2-Related CBP Activator Protein or p400 Complexes

**DOI:** 10.1155/2011/715642

**Published:** 2011-05-29

**Authors:** Tamara A. Bowman, Madeline M. Wong, Linda K. Cox, Joseph J. Baldassare, John C. Chrivia

**Affiliations:** ^1^Department of Pharmacological and Physiological Science, Saint Louis University School of Medicine, 1402 South Grand Boulevard, Saint Louis, MO 63104, USA; ^2^Laboratory of Receptor Biology and Gene Expression, National Cancer Institute, National Institute of Health Bethesda, MD 20892, USA

## Abstract

The p400 and SRCAP (Snf2-related CBP activator protein) complexes remodel chromatin by catalyzing deposition of histone H2A.Z into nucleosomes. This remodeling activity has been proposed as a basis for regulation of transcription by these complexes. Transcript levels of *p21* or *Sp1* mRNAs after knockdown of p400 or SRCAP reveals that each regulates transcription of these promoters differently. In this study, we asked whether deposition of H2A.Z within specific nucleosomes by p400 or SRCAP dictates transcriptional activity. Our data indicates that nucleosome density at specific *p21* or *Sp1* promoter positions is not altered by the loss of either remodeling complex. However, knockdown of SRCAP or p400 reduces deposition of H2A.Z*∼*50% into all *p21* and *Sp1* promoter nucleosomes. Thus, H2A.Z deposition is not targeted to specific nucleosomes. These results indicate that the deposition of H2A.Z by the p400 or SRCAP complexes is not sufficient to determine how each regulates transcription. This conclusion is further supported by studies that demonstrate a SRCAP_ΔATP_ mutant unable to deposit H2A.Z has similar transcriptional activity as wild-type SRCAP.

## 1. Introduction

The histone variant H2A.Z has been shown to have multiple functions in mammalian cells. It is essential for embryonic development, proper segregation of chromosomes [1, 2], and a number of studies indicate it plays a role in both activation and repression of transcription [3–5]. Aberrant H2A.Z expression may also play a role in some human diseases, since it has been demonstrated to play a role in cardiac hypertrophy [6] and H2A.Z levels are elevated in breast cancer [7, 8].

Recent studies have examined the genomewide distribution of H2A.Z in human cells. These studies found that nucleosomes located in promoter regions are highly enriched in H2A.Z, indicating a positive correlation that exists between gene activity and H2A.Z deposition [9, 10]. The deposition of H2A.Z at these sites has been hypothesized to promote organization of nucleosomes at the promoter providing the optimal architecture for activation of transcription. How incorporation of H2A.Z into nucleosomes functions to increase promoter organization and contribute to regulation of transcription is not clear. Several studies indicate that nucleosomes comprised of recombinant H2A.Z or native chicken erythrocyte H2A.Z are more stable than H2A-containing nucleosomes [11, 12]. Other studies, however, have raised the possibility that in certain circumstances, when nucleosomes also contain the histone variant H3.3, H2A.Z decreases nucleosome stability [13, 14]. These studies remain controversial since *in vitro* studies have reported no differences that exist in the stability of nucleosomes containing H3.3/H2A.Z compared to those containing H3.3/H2A [15].

In mammalian cells, SRCAP and p400 are the catalytic subunits of larger complexes that have been demonstrated *in vitro* and *in vivo* to deposit H2A.Z into nucleosomes [3, 5, 16]. A comparison of the structure of p400 and SRCAP indicates they share a conserved bipartite ATPase domain and a HSA domain. They also have distinct domains: SRCAP contains multiple A/T hook domains in the C terminal end, whereas p400 has a SANT domain. The human SRCAP complex contains ten subunits (SRCAP, DMAP1, BAF53a, ARP6, Gas41, Tip49a, Tip49b, ZnF-HIT1, YL1, and H2A.Z). The p400 complex shares some of these subunits (DMAP1, BAF53a, ARP6, Gas41, Tip49a, Tip49b, and YL1) but contains additional unique subunits (TRRAP, p400, TRCp120, EPC, EPC-like, TIP60, ING3, MRG15, MRGX, and MRGBP FLJ11730) [17, 18]. The presence of two complexes with H2A.Z deposition activity is intriguing and raises the possibility that they are targeted to different sites within the same promoter or to different promoters. Targeting of the p400 complex has been studied; p400 appears to be recruited to sites that bind p53 or c-Myc [5]. Specific sequences which recruit the SRCAP complex to promoters have not been established, however, interaction of SRCAP with CBP may allow targeting to a variety of promoters [19]. One outcome of targeting of the p400 and SRCAP complexes to different sites within promoters is that they may direct deposition of H2A.Z into distinct subpopulations of nucleosomes and, as a consequence, have different effects on transcription. In support of this hypothesis, recent studies on the *p21* promoter suggest that while knockdown of SRCAP or p400 expression disrupts H2A.Z deposition, only the loss of p400 results in activation of transcription [5].

In this report, we test the hypothesis that the p400 and SRCAP complexes play distinct roles in regulating transcription of the *p21* promoter through deposition of H2A.Z into distinct populations of nucleosomes. For these studies, we measure the density of H2A.Z-containing nucleosomes within the *p21* promoter and ask whether knockdown of p400 or SRCAP expression disrupts H2A.Z deposition into nucleosomes at specific locations. The results of these studies indicate the loss of p400 or SRCAP results in equivalent changes in H2A.Z binding at all nucleosomes, suggesting a non-H2A.Z-related activity associated with the p400 complex is critical to its ability to repress transcription of the *p21* promoter [20]. Studies with the *Sp1* promoter demonstrate that knockdown of p400 decreases H2A.Z deposition at all nucleosomes but did not decrease transcription. In contrast, knockdown of SRCAP decreases H2A.Z deposition at most, but not all, nucleosomes, yet transcription decreases. Thus, non-H2A.Z-related activity associated with the SRCAP complex is critical for activation of transcription at the *Sp1* promoter. Collectively, these studies indicate that H2A.Z deposition activity by the p400 and SRCAP complexes is not sufficient to explain how they regulate transcription of the *Sp1* and *p21* promoters.

## 2. Results

Previous studies in U2OS cells indicated that knockdown of p400 and SRCAP results in equivalent loss of H2A.Z deposition [5] at the *p21* promoter. Surprisingly, loss of p400 expression resulted in activation of transcription of the *p21* promoter whereas knockdown of SRCAP had no effect [5]. We subsequently confirmed these results in the lung adenocarcinoma A549 cell line (Figures 3(b) and S1 of the supplementary material available online at doi:10.1155/2001/715642). To understand how the p400 and SRCAP complexes differentially regulate transcription of the *p21* promoter we carried out a series of experiments to determine if they deposit H2A.Z into distinct nucleosomes at distinct locations within the *p21* promoter. 

Our initial studies were carried out to measure the density of nucleosomes at the *p21* promoter. For these studies, chromatin was cross-linked by treatment with formaldehyde and extensively digested by treatment with micrococcal nuclease. 

 Nondigested chromatin was removed by centrifugation and the supernatant containing released chromatin layered on a sucrose density gradient to allow separation of mononucleosomes from higher-order nucleosomes. Analysis of the fractions from the sucrose density gradient for DNA and histone H3 content validates that the preparation contains a single population of nucleosomes with DNA~147 base pairs (bp) in size (supplemental Figure  S2). This indicates that our protocol generates a pool of mononucleosomes that serves as a source of DNA to measure the nucleosomes within the *p21* promoter. For these experiments fourteen primer sets were designed that flanked the p400-binding site in the *p21* promoter that was previously characterized as overlapping the site of highest deposition of H2A.Z and the major binding site of p53 [5]. The design of the primer sets ensures amplification of overlapping regions smaller than an individual nucleosome.

**Figure 1:**
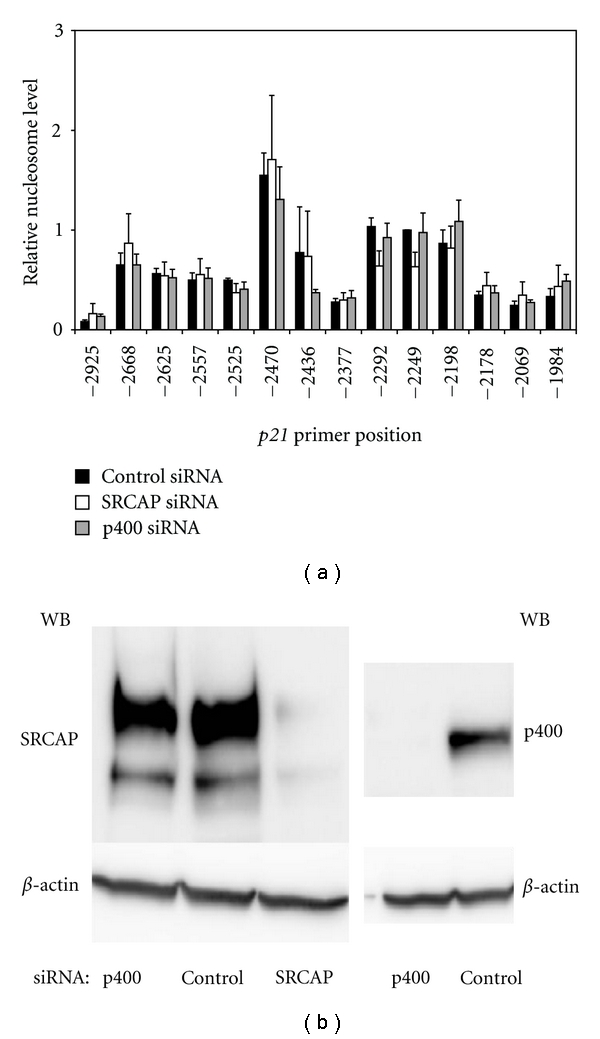
The density of nucleosomes at the *p21* promoter nucleosome is not altered in the absence of SRCAP or p400. A549 cells were transfected with control, SRCAP or p400 siRNA and harvested 72 hours later. In (a), DNA was isolated from mononucleosomes and amplified by qPCR using overlapping primers tiling the *p21 *promoter (see Table  S1(c)) and presented relative to the amount of DNA amplified at position −2249. The graph represents the mean result and standard error of three or more independent ChIP experiments. In (b), knockdown of SRCAP or p400 protein, compared to control-transfected cells, was confirmed by Western blot. Beta actin was used as a loading control.

The results of this approach indicate that the regions of highest mononucleosome DNA density overlaps the same DNA sequence where the highest p400 binding was observed in U2OS cells, from approximately-2668 to-2092 bp upstream of the transcription start site (TSS) (Figure 1(a), black bars). To ask if the nucleosomes are repositioned in the absence of SRCAP or p400, the expression of each protein was reduced by siRNA treatment. Knockdown of SRCAP or p400 was confirmed by Western blot (Figure 1(b)) and did not significantly alter nucleosome density at any position (open and gray bars in Figure 1(a)).

**Figure 2:**
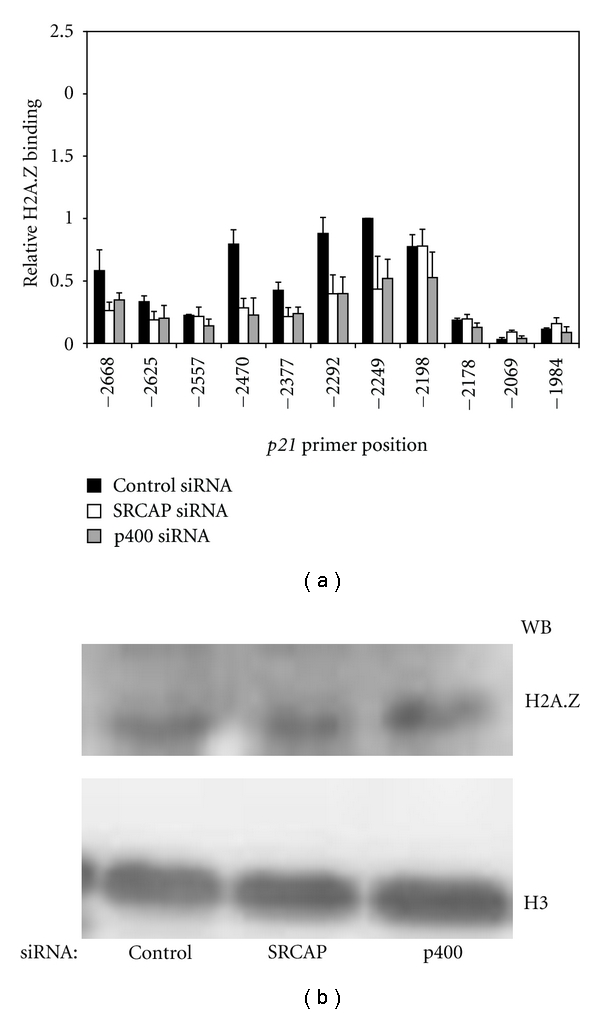
Knockdown of SRCAP or p400 decreases H2A.Z deposition into *p21* promoter nucleosomes. A549 cells were transfected with control, SRCAP or p400 siRNA and harvested 72 hours later. In (a), nucleosome ChIP assays were performed using anti-H2A.Z antibody and immunoprecipitated DNA was amplified by qPCR using the indicated primer sets (see Table  S1(c)). The amount of DNA amplified at each position is presented relative to the amount of DNA amplified at position −2249. The graph represents the mean result and standard error of three or more independent ChIP experiments. In (b), histones were acid-extracted and protein levels were determined by Western blot analysis using anti-H2A.Z antibody. Histone H3 was used as a loading control.

To determine which nucleosomes contain H2A.Z, nucleosome ChIP assays were performed using anti-H2AZ antibodies. The result of these experiments indicates that H2A.Z is not deposited into specific nucleosomes but rather is broadly distributed into all the nucleosomes adjacent to the p400: binding site in the *p21* promoter (Figure 2(a), black bars). Knockdown of either SRCAP or p400 expression results in ~50% decrease in deposition of H2A.Z into most nucleosomes (open and gray bars in Figure 2(a)). This was not due to an indirect affect, for example, decreased cellular level of H2A.Z, but due rather to loss of deposition by p400 and SRCAP (Figure 2(b)). 

**Figure 3:**
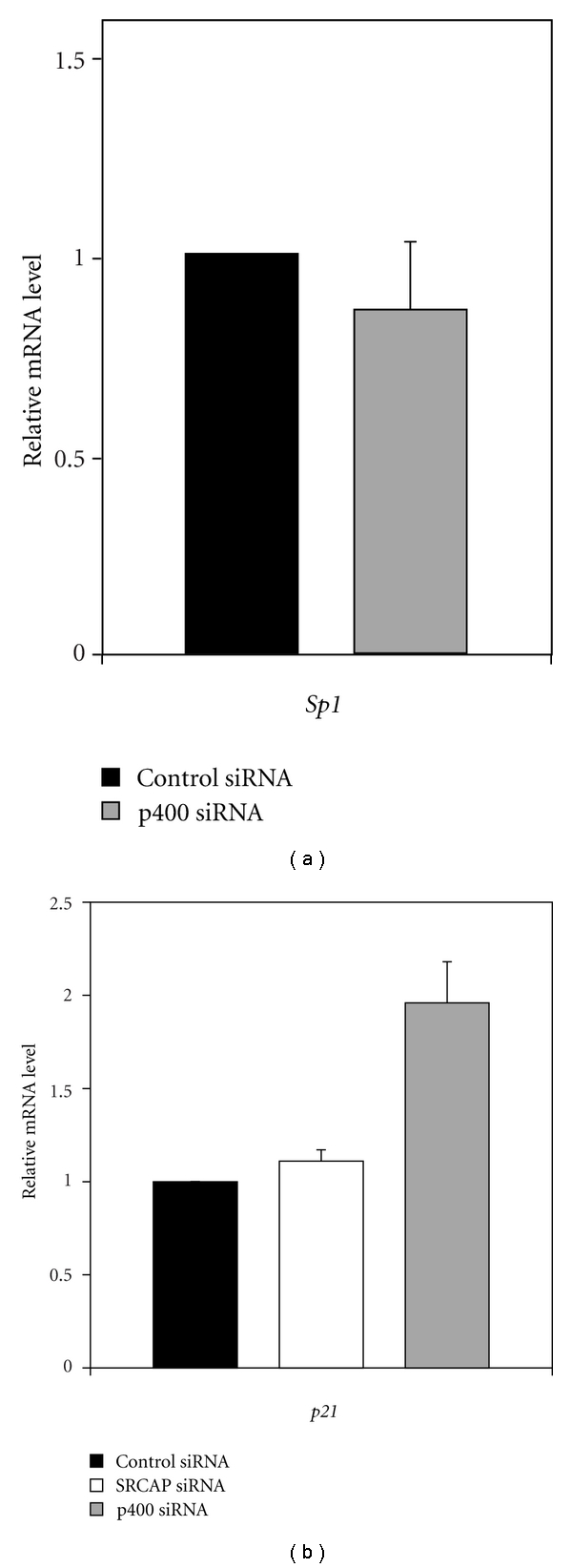
Knockdown of p400 and SRCAP expression differently regulates transcription of the *Sp1 *promoter and *p21* promoter. A549 cells were transfected where indicated with control, SRCAP or p400 siRNA, harvested 72 hours later and total RNA isolated. In (a), the level of *Sp*1 mRNA was assessed using RT-qPCR using primers listed in [3]. In (b), the level of *p21* mRNA was determined by RT-qPCR using primers listed in Table  S1(a). The graphs show the mean result and standard error from three experiments.

**Figure 4:**
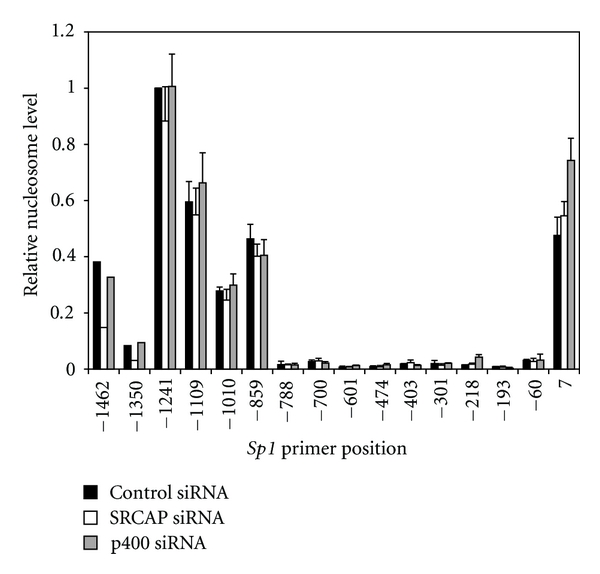
The density of nucleosomes at the *Sp1* promoter is not altered in the absence of SRCAP or p400. A549 cells were transfected with control, SRCAP or p400 siRNA and harvested 72 hours later. DNA was isolated from mononucleosomes and amplified by qPCR using overlapping primer sets tiling the *Sp1 *promoter (see Table  S2) and presented relative to the amount of DNA amplified at position −1241. The graph represents the mean result and standard error of three or more independent ChIP experiments.

To determine whether H2A.Z plays a similar role at other promoters, we also examined H2A.Z deposition into nucleosomes at the *Sp1* promoter. This promoter was chosen because knockdown of SRCAP and p400 affects transcription of *Sp1 *differently than *p21*. At the *Sp1* promoter, knockdown of SRCAP decreases transcription [3], whereas loss of p400 has no effect on transcription (Figure 3). To measure nucleosome density, sixteen primer sets were designed, flanking the SRCAP-binding site in the *Sp1* promoter that was previously characterized [3]. Examination of the *Sp1* promoter indicates that it contains nucleosomes at several positions including directly downstream of the TSS (Figure 4, black bars). This result is consistent with the findings of a recent genomewide survey of human promoters that indicate that several strongly phased nucleosomes flank the TSS of most expressed genes [10]. The *Sp1* promoter, however, also contains a large nucleosome free region (NFR) at −788 to −60, where SRCAP binds the promoter. Knockdown of p400 or SRCAP expression did not alter formation of the NFR nor did it alter the density of nucleosomes at any position (gray and open bars in Figure 4).

**Figure 5:**
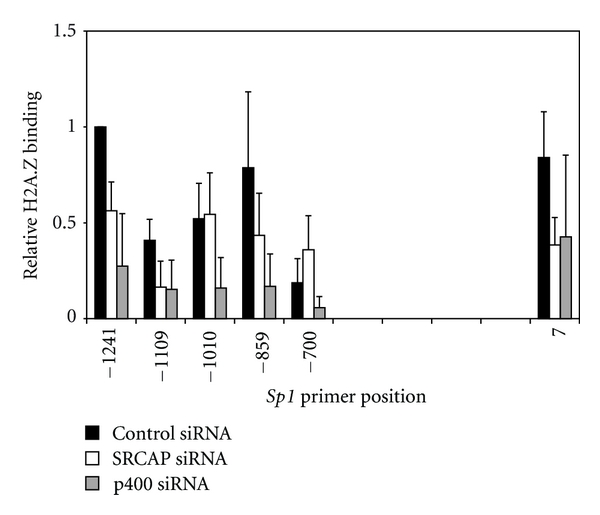
SRCAP and p400 regulate H2A.Z deposition equivalently at *Sp1* promoter nucleosomes. 549 cells were transfected with control, SRCAP, or p400 siRNA and harvested 72 hours later. Mononucleosome ChIP assays were performed using anti-H2A.Z antibody and immunoprecipitated DNA was amplified by qPCR using the indicated primer sets (see Table  S2). The amount of DNA amplified at each position is presented relative to the amount of DNA amplified at position −1241. The graph represents the mean result and standard error of three or more independent ChIP experiments.

Nucleosomes flanking the NFR in the *Sp1* promoter contained H2A.Z (Figure 5, black bars) as found at other eukaryotic gene promoters [14, 21, 22]. Knockdown of p400 or SRCAP expression did not cause depletion of H2A.Z at specific nucleosomes (gray and open bars in Figure 5), but as was observed with the *p21* promoter, p400 and SRCAP appear to have equivalent roles in maintaining normal levels of H2A.Z deposition into all nucleosomes except at positions −1010 and −700. At these positions, p400, but not SRCAP, regulates deposition of H2A.Z, indicating specificity in the selection of nucleosomes targeted by the SRCAP complex for deposition of H2A.Z. 

**Figure 6:**
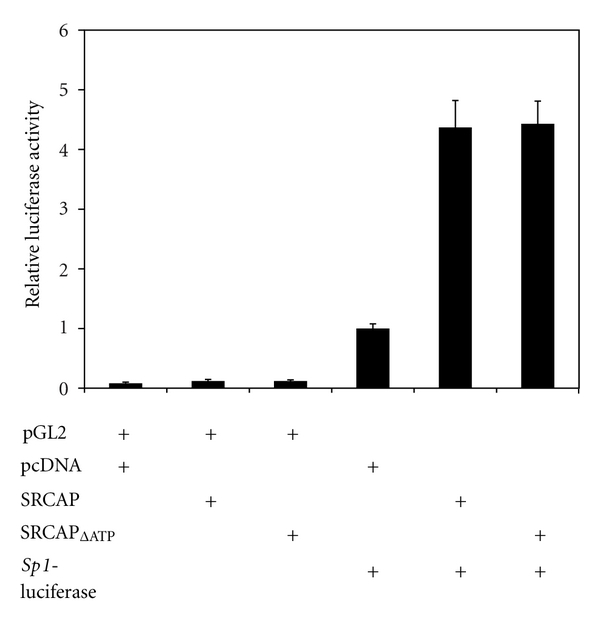
SRCAP mediates transcriptional activity independent of H2A.Z deposition. HeLa cells were transiently transfected with 300 *μ*g of *Sp-1*-luciferase reporter gene plasmid or the control pGL2 luciferase reporter gene plasmid and, where indicated, with 1000 *η*g of plasmid expressing wild-type SRCAP or the SRCAP_∆ATP_ mutant or the control vector pcDNA 3.1. The relative luciferase activity is reported compared to the luciferase activity observed in cells transfected with pGL2- luciferase and pcDNA 3.1. The graph represents the mean result and standard error of three or more independent transfection experiments.

This suggests that retention of H2A.Z deposition at specific nucleosomes following knockdown of SRCAP, but not p400, may cause repression of transcription. A more likely explanation, and one we favor, is that the ability of the SRCAP complex to regulate transcription at the *Sp1* promoter must be determined by non-H2A.Z-related activities absent in the p400 complex. To test for the presence of non-H2A.Z-related transcriptional activity of SRCAP, a mutant unable to bind ATP (K689R, SRCAP_ΔATP_) [23] and deposit H2A.Z [16] was tested for the ability to activate transcription. The result of this experiment (Figure 6) indicates that the SRCAP_∆ATP_ mutant has similar transcriptional activity as wild-type SRCAP. This indicates that SRCAP has the ability to activate transcription independent of the ability to deposit H2A.Z. 

## 3. Discussion

The ability of the SRCAP and p400 complexes to deposit the histone variant H2A.Z into nucleosomes has been previously established *in vitro* using highly purified complexes. The physiological relevance of this activity *in vivo* has been confirmed by ChIP assays that demonstrate that knockdown of SRCAP or p400 expression decreases overall H2A.Z deposition at promoters. Because of these collective observations, the role of the p400 and SRCAP complexes in regulating transcription has largely been attributed to the ability of each complex to deposit H2A.Z at promoters. In the case of the *p21* promoter, loss of H2A.Z deposition or knockdown of p400 (and subsequent loss of H2A.Z binding) results in activation of transcription. However, knockdown of SRCAP had equivalent effects on H2A.Z deposition as that seen with p400 knockdown, but did not increase transcription of the *p21* promoter. An interesting hypothesis raised by this finding is that deposition of H2A.Z at the *p21* promoter is not equivalent but is targeted by p400 or SRCAP to a distinct subset of nucleosomes.

 In this report the loss of p400 or SRCAP resulted in ~50% decrease in deposition of H2A.Z into the same nucleosomes at *p21* promoter. This redundancy may result from an overlap in the nucleosomes targeted for H2A.Z deposition or may result from redistribution of H2A.Z-containing nucleosomes from unique sites where p400 or SRCAP mediates H2A.Z deposition to new positions across the promoter. In support of the latter, incorporation of H2A.Z has been reported to increase the mobility of nucleosomes [24]. Interestingly, knockdown of p400 or SRCAP did not effect the deposition of H2A.Z at several positions within the *p21* promoter, for example, positions −2557 and −2178. This suggests that a third novel mechanism may exist for deposition of H2A.Z into some nucleosomes. Alternatively, the level of H2A.Z at any position is likely to result from equilibrium between two processes, H2A.Z incorporation and nucleosome turnover. It is therefore possible that despite the lack of p400 or SRCAP, the low rate of turnover of nucleosomes at these positions prevents a loss of H2A. Z. 

At the *Sp1* promoter while loss of p400 also decreased H2A.Z deposition into all nucleosomes, the loss of SRCAP decreased H2A.Z deposition into all nucleosomes except those at positions −1010 and −700. This observation suggests that in at least some promoters the method of H2A.Z deposition is a critical feature in maintaining H2A.Z deposition into some nucleosomes. 

The presence of a large NFR was also noted in the *Sp1* promoter region spanning positions −788 to −60 (Figure 4). Previous ChIP studies indicate these same regions are largely devoid of trimeH3K4, H2A.Z, and RNAPII and define the binding site for SRCAP [3]. Why SRCAP binds to this nucleosome free site is unclear but open chromatin may be required to allow binding of transcription factors that interact with CBP, which subsequently recruits SRCAP [19]. At other promoters, however, for example, FAD synthetase promoter, the binding sites for SRCAP and sites of H2A.Z directly coincide [3] suggesting other mechanisms might target the SRCAP to specific promoter sites.

Studies in *S. cerevisiae* have indicated that H2A.Z plays a role in nucleosome positioning and occupancy spanning the initiator region of the *GAL1* gene [25]. This suggests that promoters enriched in H2A.Z have defined nucleosome locations compared with promoters that are not significantly enriched in H2A.Z. In addition, several studies indicate that H2A.Z containing chromatin is enriched in remodeling complexes (Swi/SNF, ISWI, and CHD) that facilitate movement of nucleosomes [26]. In support of this notion, recent studies also indicate that the presence of H2A.Z in nucleosomes facilitates remodeling activity by the ISWI family members. In contrast to the expectation raised by these observations, we found that despite the ~50% decrease in deposition of H2A.Z following loss of p400 or SRCAP, the density of nucleosomes at the *p21 *or* Sp1* promoters is not altered. This suggests that at least at these promoters, the presence of H2A.Z does not facilitate chromatin-remodeling activity. One possible explanation for this observation is that H2A.Z is a minor component of nucleosomes at these promoters and hence, cannot play a major role in determining remodeling activity or chromatin structure. A second possibility is a large percentage of the nucleosomes contain H2A.Z but the replacement with H2A does not have a significant role in determining nucleosome stability, remodeling, or overall chromatin structure. Either of these scenarios is more consistent with the role of H2A.Z as a signaling molecule in which recruitment of general transcription factors is enhanced. Studies in human breast cancer cells suggest that p400-mediated deposition of H2A.Z at the estrogen receptor alpha-regulated gene, *TFF*, recruits FoxA1 to facilitate gene expression upon estrogen signaling [27]. This is further supported by studies in *S. cerevisiae* which demonstrate that H2A.Z-containing nucleosomes recruit Pol II and TBP to gene promoters [28, 29]. The C-terminal region of H2A.Z is critical for this activity and has been shown to function as an activating domain [30]. Substitution of this region by the equivalent H2A region does not rescue a loss of function mutation [28] nor does it rescue lethality in *Drosophila melanogaster *[31]. An acidic patch present in the C-terminal region of H2A.Z compared to the H2A region likely facilitates these functions [32].

Although H2A.Z is important for transcription, a clear conclusion from these studies is that deposition of H2A.Z at the *p21* and *Sp1* promoters is not sufficient to explain the transcriptional activities of the p400 and SRCAP complexes. Recent evidence indicates the role each complex plays in regulating transcription is likely due to the structural differences between SRCAP and p400. The most notable difference is the presence of a SANT domain in p400, which is absent in SRCAP. The SANT domain of p400 has recently been shown to bind directly to the histone acetyltransferase domain of TIP60. This blocks enzymatic activity and the coactivator function of TIP60 in regulating basal *p21 *gene expression via acetylated p53 [19]. Thus, loss of p400 activity mediates increased *p21 *gene expression by two mechanisms: loss of TIP60 inhibition and loss of H2A.Z deposition. In the case of the *Sp1* promoter, despite loss of H2A.Z deposition, loss of p400 expression did not alter transcription, suggesting that TIP60 does not play a critical role at this promoter. SRCAP also appears to use several mechanisms to regulate transcription. It also binds to the histone acetyltransferase CREB-binding protein (CBP) through a large spacer domain located between ATPase motifs IV and V that is not present in p400. In addition, CBP and SRCAP function synergistically to activate transcription [19]. The ability of SRCAP to activate transcription of the *Sp1* promoter but not the *p21* promoter may be dependent on its ability to serve as a platform for recruitment of CBP. Consistent with the role of SRCAP to function as a recruitment platform, we found that the SRCAP_∆ATP_ mutant retained the ability to activate transcription of the *Sp1* promoter despite its inability to deposit H2A.Z. This mutant is not able to function in a transgenic fly model, implying that both the H2A.Z deposition and scaffolding activities of SRCAP are critical for its normal function *in vivo*. These results suggest that the deposition of H2A.Z does contribute to, but is not sufficient to describe, the transcriptional activity of SRCAP or p400.

In summary, our studies demonstrate that the p400 and SRCAP remodeling complexes have overlapping redundancy in targeting and deposition of H2A.Z into promoter nucleosomes. In addition, we demonstrate that while H2A.Z deposition may be a critical activity of these complexes, it is not sufficient to explain the effect the p400 and SRCAP complexes have on transcription of the *Sp1* and p21 promoters.

## 4. Materials and Methods

### 4.1. Antibodies

The anti-SRCAP affinity-purified rabbit polyclonal antibody was generated against SRCAP as described in [15] and the anti-p400 antibody was raised in rabbits against the p400 C-terminal peptide (SSDSPSQQPKLQMRVPAVRLKTPTKPP). Other commercial antibodies were histone H2A.Z polyclonal (Abcam, ab4174), histone H3 polyclonal (Abcam, ab18262), and mouse monoclonal anti-*β*-actin antibody (Sigma, A5441). 

### 4.2. Cell Culture

The human lung adenocarcinoma cell line, A549, and human cervical carcinoma, HeLa, (ATCC) were cultured in Dulbecco's modified Eagle's medium (Life Technologies) supplemented with 10% fetal bovine serum (Sigma) and 1% penicillin/streptomycin (Life Technologies).

### 4.3. Reverse Transcription Quantitative Real-Time PCR (RT-qPCR)

Total cellular RNAs were extracted with TRIzol reagent (Life Technologies) according to the manufacturer's protocol. Reverse transcription reactions were done as described in lab protocols [3] on 4 *μ*g of total RNAs, oligo-dT (Promega), and SuperScript II Reverse Transcriptase (Life Technologies) according to the manufacturer's protocol. cDNA levels were measured by RT-qPCR as in qPCR protocol with the following exceptions: the **β*-actin* annealing temperature was 52°C for 30 s and quantification for 40 cycles, the *p21* annealing temperature was 60°C for 30 s and quantification for 40 cycles. Primer sequences for *p21 *are listed in the Table  S1(b).

### 4.4. Mononucleosome Preparation

 Chromatin cross-linking and nuclei preparation was performed as described in [3]. The nuclei were resuspended in 500 *μ*L of 0.32 M sucrose, 10 mM HEPES, pH 7.9, 60 mM KCl, 2 mM EDTA, 10 mM sodium butyrate, 0.5 mM DTT, 1 mM PMSF, and supplemented with protease inhibitors (Roche Applied Science) and layered on top of 500 *μ*L of 30% sucrose 10 mM HEPES, pH 7.9, 60 mM KCl, 2 mM EDTA, 10 mM sodium butyrate, 0.5 mM DTT, 1 mM PMSF supplemented with protease inhibitors (Roche Applied Science) and centrifuged at 500 xg for 5 minutes at 4°C. The pelleted nuclei were washed with micrococcal nuclease buffer (10 mM HEPES, pH 7.9, 60 mM KCl, 15 mM NaCl, and 0.34 mM sucrose) and resuspended in micrococcal nuclease buffer plus 10 mM sodium butyrate, 3 mM CaCl_2_, mM 0.5 mM DTT, and 0.6 Kunitz units of micrococcal nuclease (New England BioLabs) per microgram DNA at 37°C in a water bath for 11 minutes. The digestion was stopped on ice with the addition of EGTA (10 mM) and nondigested chromatin was removed by centrifugation at 11,000 ×g for 10 minutes at 4°C. A portion of the supernatant was analyzed by fractionation on a sucrose gradient to determine the extent of digestion. A second portion was processed by Western blot analysis to verify that histones were present in the same DNA fraction containing mononucleosomes. A third portion was used to determine nucleosome density.

### 4.5. Quantitative Real-Time PCR (qPCR)

The amount of DNA corresponding to specific regions of the *p21* and *Sp1* promoters present in the mononucleosome DNA preparations or in the nucleosome ChIP eluates was measured by quantitative real-time PCR (DNA Engine Opticon 2 System, Bio-Rad) with 2x fastStart SYBR Green master Mix (Roche Applied Science) according to protocols developed in our lab [3]. The reaction mixture consisted of 500 *η*M of forward and reverse primer (see Tables S1(c) and S2), mononucleosomal DNA and the SYBR Green Master Mix (Roche Applied Science). The qPCR protocol was: 95°C for 5 minutes followed by three-step amplification (denaturation 95°C, 30 seconds; annealing 60°C, 30 seconds; extension 72°C, 40 seconds) and quantification of DNA for 35 cycles.

To accurately determine the amount of promoter DNA within the nucleosome DNA sample, the standard curve method is used as described [3]. In this method, for each primer set, a series of amplification curves are generated using known amounts of genomic DNA (not nucleosome DNA). The equation for PCR kinetics is *N*_Ct_ = *N*_O_×(eff)^Ct^, where *N*_O_ is the initial amount of DNA in the sample, *N*_Ct_ is the amount of DNA at the threshold cycle, and eff is the PCR efficiency. This equation can be converted into a linear form Ct = [−1/Log  (eff)] × Log  *N*_*O*_ + Log  (*N*_Ct_)/Log  (eff). Therefore, a linear standard curve with a slope =[−1/Log (eff)] can be constructed by plotting Ct values against the Log N_O_ of the standards. As standards we use 10, 1, and 0.1 ng of genomic DNA. DNA obtained from nucleosomes is not used as a standard, since it lacks promoter regions digested by micrococcal nuclease. For both the sample and standard curve reactions we typically obtain PCR efficiency of greater than 1.9 (95%).

### 4.6. Knockdown of SRCAP or p400

Knockdown transfections were carried out according to lab protocols [3]. Cells were transfected using Dharma*FECT1 *(Dharmacon) siRNA transfection reagent according to the manufacturer's protocol to transfect A549 using 40nM of the Dicer substrate siRNA targeting: control, SRCAP, or p400 (see Supplemental Table  S3, all siRNA came from IDT). Cells were harvested 72 hours after transfection for protein analysis, mononucleosomes preparation or RNA isolation.

### 4.7. Chromatin Immunoprecipitation

ChIP assays were performed according to the protocol developed in our lab [3].

### 4.8. Nucleosome Chromatin Immunoprecipitation

Mononucleosomes (6 *μ*g DNA) were diluted to a final volume of 1 mL with Sonication buffer and precleared using 100 *μ*L of protein A/G-agarose beads (50% slurry) in PBS. The supernatant was cleared a second time using 200 *μ*L of protein A/G beads (50% slurry) that had been preblocked with nonfat milk (1%), BSA (0.1%) and normal rabbit IgG. The cleared ChIP lysate was then incubated for 18 hours with 12 *μ*g anti-H2A.Z antibody or 12 *μ*g normal rabbit IgG followed by additional 2-hour incubation with 50 *μ*L of protein A/G beads (50% slurry). The ChIP eluate was then obtained by washing the protein A/G beads and eluting the bound DNA as described above. Following reversal of cross-linking, protein digestion, and DNA purification, the DNA was resuspended in 100 *μ*L dH_2_O. 

### 4.9. Acid Extraction of Histones

A 459 nuclei were lysed in 5 volumes of 25 mM HEPES (pH 7.8), 1.5 mM MgCl_2_, 10 mM KCl, 0.1% Nonidet P-40, 10 mM sodium butyrate, 0.5 mM DTT, 1.5 mM PMSF, protease inhibitors (Roche Applied Science) and hydrochloric acid (0.2 M final) for 30 minutes on ice. Sample was centrifuged 11,000 g for 10 minutes at 4°C. The supernatant was dialyzed against 200 mL acetic acid (0.1 N), twice for 1 hour each then against 200 mL dH_2_O for 1 hour, 3 hours, and overnight. The sample was lyophilized to concentrate the proteins. 

### 4.10. Transfection

HeLa cells were transfected with 300 *μ*g of the *Sp1*-*luciferase* or pGL2-*luciferase* expression plasmid and 1000 *η*g of the plasmids expressing either SRCAP or SRCAP_∆ATP_. The *Sp1-luciferase* plasmid was constructed by subcloning *Sp1* promoter DNA (−1241 to + 100) into the KpnI and ZhoI sites of the pGL2 basic luciferase plasmid (Promega). The *SRCAP* (1-2971) or SRCAP_∆ATP_ (K649R) expression plasmids were constructed as described [18, 24]. Each transfection was adjusted to contain equal molar amounts of CMV promoter by use of the pcDNA3.1 Myc/His plasmid. Transfections were carried out using Lipofectamine (Life Technologies) according to the manufacturer's directions. Following an overnight incubation, cells were harvested and assayed for luciferase activity. The relative luciferase activity reported was performed in triplicate as described in [24].

## Supplementary Material

Figure S1 demonstrates by standard ChIP assay that knockdown of SRCAP reduces H2A.Z deposition at the p21 promoter.
Figure S2 demonstrates that nucleosomes prepared by micrococcal nuclease digestion are highly enriched in DNA of ^~^150 base pairs and contain histone H3, consistent with a preparation highly enriched in mononucleosomes.
Table S1(a) provides the sequence of the primer used to measure p21 mRNA levels and Table S1(b) indicates the location and sequence of primers to measure p21 promoter levels in standard H2A.Z ChIP experiments. Table S1(c) indicates the location and sequence of primers to measure p21 promoter levels in H2A.Z nucleosome ChIP experiments.
Table S2 indicates the location and sequence of primers to measure sp1 promoter levels in H2A.Z nucleosome ChIP experiments.
Table S3 provides the sequence of the p400 and SRCAP siRNAs used in the study.
