# Supplementary material for: Loss of H2A.Z Is Not Sufficient to Determine Transcriptional Activity of Snf2-Related CBP Activator Protein or p400 Complexes
Source: Int J Cell Biol. 2011 May 29;2011:715642. doi: 10.1155/2011/715642 (PMC3140016; doi:10.1155/2011/715642)
Supplement: Supplementary file 1 — Figure S1 demonstrates by standard ChIP assay that knockdown of SRCAP reduces H2A.Z deposition at the p21 promoter. Figure S2 demonstrates that nucleosomes prepared by micrococcal nuclease digestion are highly enriched in DNA of ~150 base pairs and contain histone H3, consistent with a preparation highly enriched in mononucleosomes. Table S1(a) provides the sequence of the primer used to measure p21 mRNA levels and Table S1(b) indicates the location and sequence of primers to measure p21 promoter levels in standard H2A.Z ChIP experiments. Table S1(c) indicates the location and sequence of primers to measure p21 promoter levels in H2A.Z nucleosome ChIP experiments. Table S2 indicates the location and sequence of primers to measure sp1 promoter levels in H2A.Z nucleosome ChIP experiments. Table S3 provides the sequence of the p400 and SRCAP siRNAs used in the study. [file 715642.f1.pdf]

Supplemental Figure S1: SRCAP knockdown reduces H2A.Z binding at the *p21* promoter. A schematic of the *p21* promoter and the location of primers used in the study are shown. The black bar represents the site of highest H2A.Z deposition in U2OS cells. Standard ChIP assay was used to determine the relative level of H2A.Z binding in A549 cells harvested 72 hours post-transfection with control or SRCAP siRNA. The DNA was amplified by qPCR using overlapping primers tiling the *p21* promoter, listed in Supplemental Table 1B.

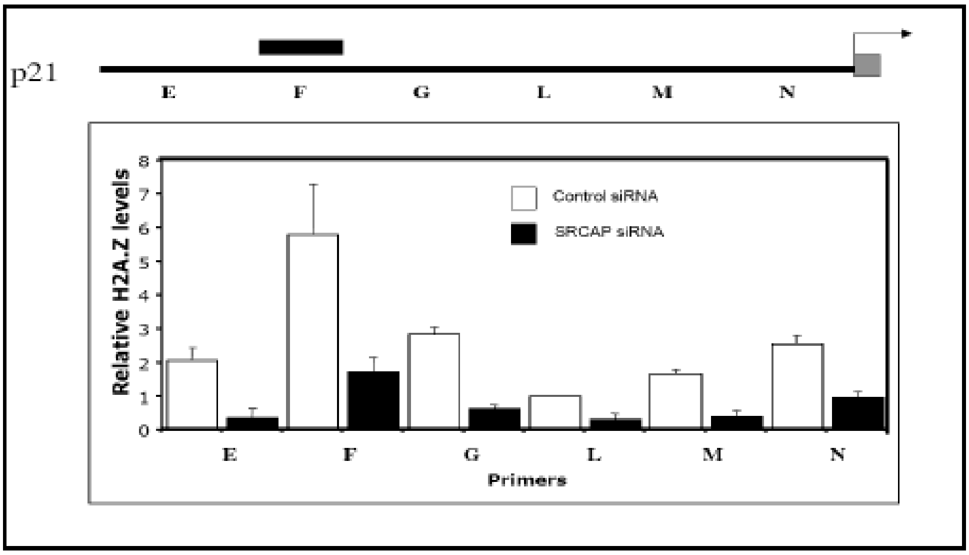

.
