# Supplementary material for: Loss of H2A.Z Is Not Sufficient to Determine Transcriptional Activity of Snf2-Related CBP Activator Protein or p400 Complexes
Source: Int J Cell Biol. 2011 May 29;2011:715642. doi: 10.1155/2011/715642 (PMC3140016; doi:10.1155/2011/715642)

Supplemental Figure S2. Sucrose Density Gradient Fractionation. Nucleosomes were prepared from A549 cells digested with micrococcal nuclease and loaded onto a 5 – 30% sucrose density gradient. In the **top panel**, a portion of each fraction was digested with Proteinase K followed by phenol-chloroform extraction and ethanol precipitation then the purified DNA was size fractionated on a 2.5% agarose gel. In the **bottom panel**, fractions from the sucrose density gradient were run on a SDS-Page and Western blotted using anti-histone H3 antibody.

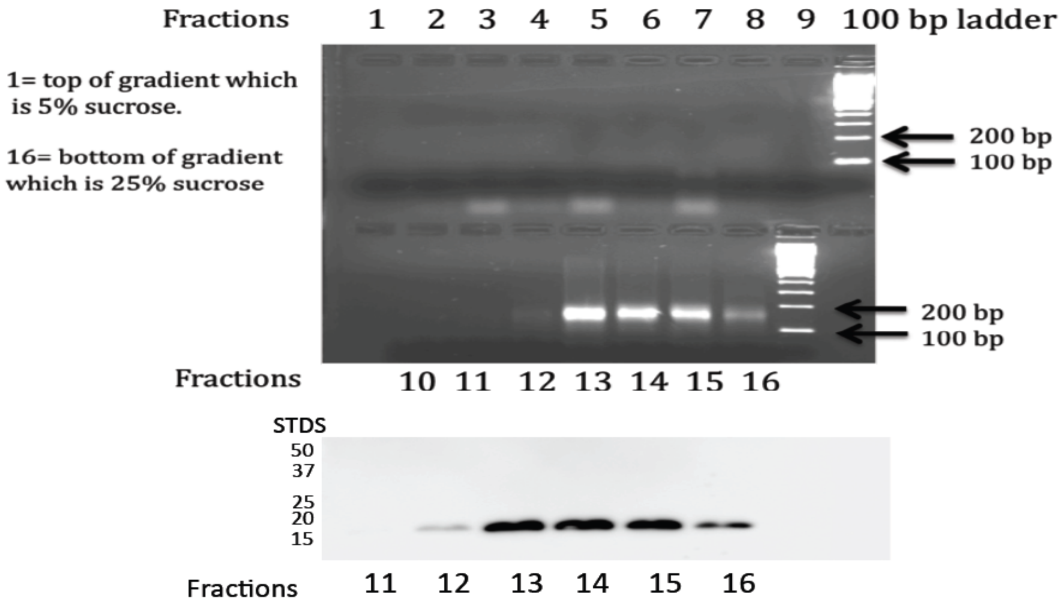

Supplement: Supplementary file 2 [file 715642.f2.pdf]
