# Supplementary material for: Loss of H2A.Z Is Not Sufficient to Determine Transcriptional Activity of Snf2-Related CBP Activator Protein or p400 Complexes
Source: Int J Cell Biol. 2011 May 29;2011:715642. doi: 10.1155/2011/715642 (PMC3140016; doi:10.1155/2011/715642)
Supplement: Supplementary file 3 [file 715642.f3.pdf]

Supplemental Table 1: *p21* primers employed in the study. **A.** RT-qPCR primers used for mRNA detection. **B** Standard ChIP primers. **C.** Nucleosome ChIP primers

A.

p21 mRNA Primer (5' – 3')	Forward: GGAGACTCTCAGGGTCGAAA<br>Reverse: GGATTAGGGCTTCCTCTTGG

B.

Primer Name	Primer Position	Sequence (5' – 3')
p21-E	-2525	ACATTGTTCCCAGCACTTCC<br>TAGGGGAATGGTGAAAGGTG
p21-F	-2292	CTGTGGCTCTGATTGGCTTT<br>CTCCTACCATCCCCTTCCTC
p21-G	-2073	GGATCTGATGCATGTGTGCT<br>CAAGGGAGCAGGCTGTTAAA
p21-L	-1034	TTGTCATTTTGGAGCCACAG<br>GGGCTCAGAGAAGTCTGGTG
p21-M	-695	CTCTCCAATTCCCTCCTTCC<br>TAGGTGCTGGAGGTGCTTCT
p21-N	-345	GGGGCTCATTCTAACAGTGC<br>GACACATTTCCCCACGAAGT

C.

Primer Position	Sequence (5'- 3')	Product size (bp)
-2925 / -2793	TGCTAGGAACATGAGCAAACTG<br>CCCGAGTAGCTGGGATTACA	133
-2668 / -2560	GGCTGCCTCTGCTCAATAAT<br>ATAGGGGCAGTCAGCTTTCA	109
-2625 / -2505	TCTGGGGTCTCACTTCTTGG<br>AGGAAGTGCTGGGAACAATG	121
-2557 / -2457	GGGACTCCCCAGTCTCTTTC<br>ATGTGAGGAAGGCTCAGTGG	101
-2525 / -2412	ACATTGTTCCCAGCACTTCC<br>TAGGGAATGGTGAAAGGTG	114
-2470 / -2364	AGCCTTCCTCACATCCTCCT<br>GACAAGAGTGCCCAGTCCAG	107
-2436 / -2322	CCTTTCACCATTCCCCTACC<br>AGGCAGCATAGGGATGGAG	115
-2377 / -2248	CTGGACTGGGCACTCTTGTC<br>GTTGGGACATGTTCCTGACG	130
-2292 / -2196	CTGTGGCTCTGATTGGCTTT<br>GCAGCCCAAGGACAAAATAG	97
-2249 / 2164	CATGTTGAGCTCTGGCATAGA<br>TCTCCTACCATCCCCTTCCT	86
-2198 / -2092	GCCTGTTTTCAGGTGAGGAA<br>CTGCTGGCAGATCACATACC	107
-2178 / -2048	AGGGGATGGTAGGAGACAGG<br>ACACAAGCACACATGCATCA	131
-2068 / -1925	CTGATGCATGTGTGCTTGTG<br>AGTTTGCAACCATGCACTTG	145
-1984 / -1848	GGGCAGAAGTCCTCCCTTAG<br>CTCACCACCACCACGACAT	137
