# Supplementary material for: Loss of H2A.Z Is Not Sufficient to Determine Transcriptional Activity of Snf2-Related CBP Activator Protein or p400 Complexes
Source: Int J Cell Biol. 2011 May 29;2011:715642. doi: 10.1155/2011/715642 (PMC3140016; doi:10.1155/2011/715642)
Supplement: Supplementary file 4 [file 715642.f4.pdf]

SupplementalTable2: *Sp1* primers employed in the study. Nucleosome ChIP

Primer Position	Sequence (5'- 3')	Product size (bp)
-1462 / -1334	CAGCAGAGGCCTCAGTTCTT<br>GATGAGGTCATGTCCTTCAGTATTT	129
-1350 / -1222	AAGGACATGACCTCATCGTGT<br>CAGCTTTTTGCAATCACGAA	129
-1241 / -1135	TTCGTGATTGCAAAAAGCTG<br>TTGAGGCTAAAGTGCGGATAA	107
-1109 / -983	AAGGGTTAGCGCCTTTGTC<br>GGGACGAGATCTGGTGACAG	127
-1010 / -885	GGTGAGTGCTGTCACCAGAT<br>TGCCCCTATAAAGTACCTCTCAA	126
-859 / -751	TAGTGTTGATGCGGAACTGC<br>TCCTCAGGGGACGTTGTTAC	109
-788 / -657	GCGTCCCACCTTAATGACTGT<br>CGCCTCCTAGTTAGCCAATG	132
-700 / -582	ATGATGGGCAGCTCCAGTAA<br>ACTTGGAGTGGCAGAGGAGA	119
-601 / -476	TCTCCTCTGCCACTCCAAGT<br>GCAGCCAGGAACGTTATGAT	126
-474 / -378	CGCCTGATTTCTGATTGGTT<br>GAATCCGGGATATGCTTGG	97
-403 / -273	ACTAGGATTTTGCCCAAGCA<br>TGATTGGCTTGGAAGGAAAC	131
-301 / -190	GGTTGACTGGTTTCCTTCCA<br>TGGTGTCCGCCTAAAAAGAC	112
-218 / -113	GAGAGGGCGGTCTTTTTAGG<br>GCAGAAGAAAGACGGTGGAA	106
-193 / -85	ACCAGGCACGCAACTTAGTC<br>CAATGAGGGAGGGGGAAA	109
-60 / +80	GGCCTAGGTTGGGCTTGT<br>AAGCTCAAGGGGGTCCTGT	139

+7 / +118	GTTCGCTTGCCTCGTCAG<br>GGACTCATCCTTACCGCTC	112
