# Supplementary material for: Loss of H2A.Z Is Not Sufficient to Determine Transcriptional Activity of Snf2-Related CBP Activator Protein or p400 Complexes
Source: Int J Cell Biol. 2011 May 29;2011:715642. doi: 10.1155/2011/715642 (PMC3140016; doi:10.1155/2011/715642)
Supplement: Supplementary file 5 [file 715642.f5.pdf]

Table 3: siRNA duplex sequences

Name	Sequence (5’ – 3’)
Control	CAUCGAGACGCUAGCAGAUCCUGCG
	GCGUAGCUCUGCGAUCGUCUAGGACGC
SRCAP	CAACAAACAGCCUUGGCAUCCAGAT
	GCGUUGUUUGUCGGAACCGUAGGUCUA
p400	CCAACGUUUCAAUUCAAGCUCCCACUU
	GUGGGAGCUUGAAUUGAAACGUUGG

Name	Sequence (5’ – 3’)

.
